# Reference gene selection to determine differences in mitochondrial gene expressions in phosphine-susceptible and phosphine-resistant strains of *Cryptolestes ferrugineus*, using qRT-PCR

**DOI:** 10.1038/s41598-017-07430-2

**Published:** 2017-08-01

**Authors:** Pei-An Tang, Jin-Yan Duan, Hai-Jing Wu, Xing-Rong Ju, Ming-Long Yuan

**Affiliations:** 1 Collaborative Innovation Center for Modern Grain Circulation and Safety, College of Food Science and Engineering, Nanjing University of Finance and Economics, Nanjing, Jiangsu 210023 China; 2 State Key Laboratory of Grassland Agro-Ecosystems, College of Pastoral Agricultural Science and Technology, Lanzhou University, Lanzhou, Gansu 730020 China

## Abstract

*Cryptolestes ferrugineus* is a serious pest of stored grain and has developed high levels of resistance to phosphine fumigants in many countries. Measuring differences in expression levels of certain ‘resistant’ genes by quantitative real-time PCR (qRT-PCR) may provide insights into molecular mechanisms underlying resistance to phosphine in *C. ferrugineus*, but reliable qRT-PCR results depend on suitable reference genes (RGs). We evaluated the stability of nine candidate RGs across different developmental stages and phosphine strains of *C. ferrugineus*, using four softwares. The results showed that *RPS13* and *EF1α* were the most stable RGs, whereas *α-TUB* was the least under developmental stages. Across the different strains, *RPS13* and *γ-TUB* were the most stable RGs, whereas *CycA* and *GAPDH* were the least. We confirmed the reliability of the selected RGs by qRT-PCR analyses of the mitochondrial *cox1* gene. Expression of *cox1* was not significantly different in the phosphine-resistant strain compared with the phosphine-susceptible strain, but three mitochondrial genes (*nad3, atp6* and *cob*) were significantly down-regulated. These results suggest that alterations in the expressions of these three genes may be associated with phosphine resistance in *C. ferrugineus*. The findings will facilitate future functional genomics studies on the development and phosphine resistance in *C. ferrugineus*.

## Introduction

The rusty grain beetle *Cryptolestes ferrugineus* (Coleoptera: Laemophloeidae) is a serious pest of stored cereal grains and processed commodities throughout the world^1, 2^. In China, *C. ferrugineus* is considered to be the most important pest of the *Cryptolestes* genus and has caused serious economic losses in several southern provinces in recent years^3^. Currently, phosphine (PH_3_) is the most widely used fumigant for controlling this species in grain stores worldwide^4^. With the continuing phase-out of methyl bromide because of its contribution to ozone depletion, it is likely that phosphine will continue to play an important role in the protection of stored products for the foreseeable future^5^. However, phosphine resistance occurs worldwide and is a major challenge to its continued effective use^4–6^. Recent studies from a variety of different countries have indicated that *C. ferrugineus* is one of the most highly resistant insect pests to phosphine^4, 5^. However, the molecular mechanisms of this resistance are currently poorly understood.

The earliest reports of resistance to phosphine in the field were made in 1972-1973^7^, but since that time, phosphine resistance has been reported in a whole host of the most common stored-product insect pests, including *Tribolium castaneum* ^8–12^, *Rhyzopertha dominica* ^8, 9, 11, 12^, *Sitophilus oryzae* ^13–15^, *Lasioderma serricorne* ^16^, *Sitophilus zeamais* ^17^, *Oryzaephilus surinamensis* ^12, 18^, *C. ferrugineus* ^4, 5^ and *Liposcelis bostrychophila* ^19^. Despite several decades of study, the biological mechanisms underlying resistance to phosphine and its toxicity are not well understood^20^. Mitochondria are broadly recognised as the intracelluluar site of biological action of phosphine, where it inhibits aerobic respiration^21^. Phosphine resistance has consistently been associated with carbon dioxide production, metabolic rate, respiration rate and even with walking activity^12, 18, 22–24^. Mitochondrial DNA is known to encode a limited number (<20) of the polypeptide components of respiratory complexes I, III, IV, and V. Genes controlling expressions of the components of complex II are conspicuously lacking in the mitochondrial genomes that have so far been characterized^25^, whilst the following genes of the mitochondrial genome are known to encode the subunits of respiratory complexes I, III, IV and V, respectively: NADH dehydrogenase subunit 3 (*nad3*), cytochrome b (*cob*), cytochrome c oxidase subunit I (*cox1*) and ATP synthase F0 subunit 6 (*atp6*). To date, we have used next generation gene sequencing techniques to establish a *C. ferrugineus* transcriptome database (GenBank accession number SRA245468) to identify potential molecular targets for the control of *C. ferrugineus*. The sequences in this database can be used in gene expression analyses to reveal the molecular mechanisms underlying biological processes such as insecticide resistance. However, despite a number of transcriptome analyses related to insecticide resistance having been performed with the aim of detecting and managing resistant insect strains, very limited information is currently available for the expression profiles of target genes associated with phosphine resistance in *C. ferrugineus*.

Quantitative real-time PCR (qRT-PCR) is a valuable tool for gene expression studies due to its high sensitivity, specificity and convenience in high throughput analyses^26^. In general, when the technique is used to analyze gene expressions, reference genes (RGs) are needed to normalize nonspecific variations or errors that can be caused by sample quantity, variations in efficiency of RNA extraction, cDNA concentration, primer performance, PCR efficiency and experimental precision^27, 28^. The optimal number of RGs is another normalization factor because one single reference gene is not enough to normalize gene expression data^28, 29^. The ideal RGs used in quantitative gene expression studies should be stable under the experimental conditions employed^27^. In the last two decades, several housekeeping genes, such as alpha-tubulin (*α-TUB*), beta actin (*β-ACT*), elongation factor 1 alpha (*EF1α*), glyceraldehydes 3 phosphate dehydrogenase (*GAPDH*), 18 S ribosomal RNA (*18 S*), ribosomal protein S13 (*RPS13*) and ribosomal protein L13a (*RPL13a*), have been widely used as RGs because they are considered to have a stable and uniform expression pattern as a result of their basic, ubiquitous, cellular functions and consistencyof expressions under different experimental conditions^26, 30^. However, it has now been shown that many of the conventional RGs (e.g. *18 S*, *β-ACT* and *GAPDH*) exhibit variable expression levels depending on the organism, its developmental stage, its response to external stimuli and the experimental conditions ^27, 30–32^. Thus, if the RGs are not selected appropriately, it could result in inaccurate calculation of the normalization factor, which could obscure the detection of actual biological differences between samples. Therefore, it is necessary to validate the expression stability of RGs under specific experimental conditions before using them for normalization. To date, many studies have been conducted in various insect groups to select suitable RGs under different experimental conditions, such as Lepidoptera^29^, Hymenoptera^33^, Diptera^34^, Corrodentia^35^, Coleoptera^28^, Homoptera^30, 36, 37^, Thysanoptera^38^ and Hemiptera^31^. However, none of these previous studies has focussed on *C. ferrugineus*, which limits the current opportunities for functional genomics studies on this important pest.

In this study, the expression stability of nine candidate RGs, namely succinate dehydrogenase complex subunit A (*SDA*), cyclin A (*CycA*), γ-tubulin (*γ–TUB*), *α-TUB*, *EF1α*, *GAPDH*, *RPL13a*, *RPS13* and *β*-*ACT*, was evaluated in different developmental stages and phosphine resistant strains of *C. ferrugineus* using four statistical algorithm methods (geNorm^39^, NormFinder^40^, BestKeeper^41^ and delta Ct^42^). The optimal number of RGs (normalization factors, NFs) was also determined. The reliability of the selected RGs was confirmed by qRT-PCR analyses of the mitochondrial *cox1* gene. The expression profiles of three other mitochondrial genes (*nad3*, *atp6* and *cob*) were aso analyzed by qRT-PCR for different phosphine resistant strains of *C. ferrugineus*. Previous studies have shown the importance of these mitochondrial genes in phospine resistance^43^. The present study sought to identify reliable RGs that could be used to accurately measure gene expressions under different developmental stages and phosphine resistant strains, which could therefore benefit future functional genomics studies of the development and phosphine resistance in *C. ferrugineus*.

## Results

### Amplification efficiency and expression profiles of the candidate RGs and target genes

The primer sets of the nine candidate RGs and four target genes for qRT-PCR used in the study were shown in Table 1. The primer specificities were validated by the presence of a single band of the expected molecular weight on a 3% agarose gel (Supplementary Fig. S1) and a sharp peak in the melting curve analysis (Supplementary Fig. S2). A standard curve was generated for each gene using five-fold serial dilutions of cDNA (Supplementary Fig. S3). The amplification efficiencies (E%) of the primer pairs ranged from 94.71% for *SDA* to 110.06% for *cox1*, with the correlation coefficient (*R* ^2^) values varying from 0.993 to 0.999. On this basis, all primers were considered suitable for use in the accurate amplification of the corresponding genes (Table 1).

**Table 1:** Gene primer sequences and amplicon characteristics.

Gene symbol	Gene name	Primer sequence (5′–3′)	Product length (bp)	Tm (°C)	E (%)	*R* ^2^
*α-TUB*	*alpha tubulin*	F: GGTCAGGCTGGTGTTCAAAT	173	60	102.77	0.996
		R: ACGATGACCACTCTGGGAAC				
*CycA*	*cyclin A*	F: TTCGAGTGCATGAAGACCAG	193	60	106.76	0.999
		R: TTGCGTTTACGGCTCTCTTT				
*EF1α*	*elongation factor 1α*	F: CCAGGCATGGTAGTGACCTT	184	60	98.05	0.997
		R: TTGGAGGGTTGTTTTTGGAG				
*γ-TUB*	*gamma tubulin*	F: TGATATTTGGGAAGGCAAGG	201	60	103.28	0.993
		R: CGCCCAAGAGCGTAATAATC				
*GAPDH*	*glyceraldehyde 3-phosphate dehydrogenase*	F: GTTCCGATGTTTGTGTGTGG	230	60	101.64	0.993
		R: TTCTGCAATCCCGATCTACC				
*RPL13a*	*ribosomal protein L13a*	F: GCTCGTGGTCCATTTCATTT	240	60	103.71	0.998
		R: TCCCACTTCATGTGACAAGC				
*RPS13*	*ribosomal protein S13*	F: ATCCGTAAGCATTTGGAACG	162	60	102.48	0.999
		R: AGCCACTAAGGCTGAAGCTG				
*SDA*	*succinate dehydrogenase complex subunit A*	F: GCCAATTCCTGTTCTTCCAA	155	60	94.71	0.994
		R: CTCCGTGAACTGAAGCACAA				
*β-ACT*	*beta actin*	F: CCTCTTCCAGCCTTCCTTCT	248	60	98.63	0.997
		R: CACCGATCCAGACGGAGTAT				
*nad3*	*NADH dehydrogenase subunit 3*	F: GCCTTTTGAATGTGGATTTGA	119	60	97.11	0.998
		R: TTGGGAATAAAAGGGTAATTTCT				
*atp6*	*ATP synthase F0 subunit 6*	F: TTTCTAGGATTATTCCCCTATATTTTT	129	60	109.60	0.999
		R: GGCTAATATATGGGTTGTATTATTGAT				
*cob*	*Cytochrome b*	F: AAAATTTTTAAGAACACAATTCTACCC	100	60	104.75	0.998
		R: AACAGGGCGAGCTCCAAT				
*cox1*	*Cytochrome c oxidase subunit I*	F: TACCGGGGTTGTTCTAGCTG	232	60	110.06	0.993
		R: AAAATGTTGGGGGAAAAAGG				

Tm: melting temperature; bp: base pairs; *E*: efficiency of primer; *R* ^2^: regression coefficient.

**Figure 1:**
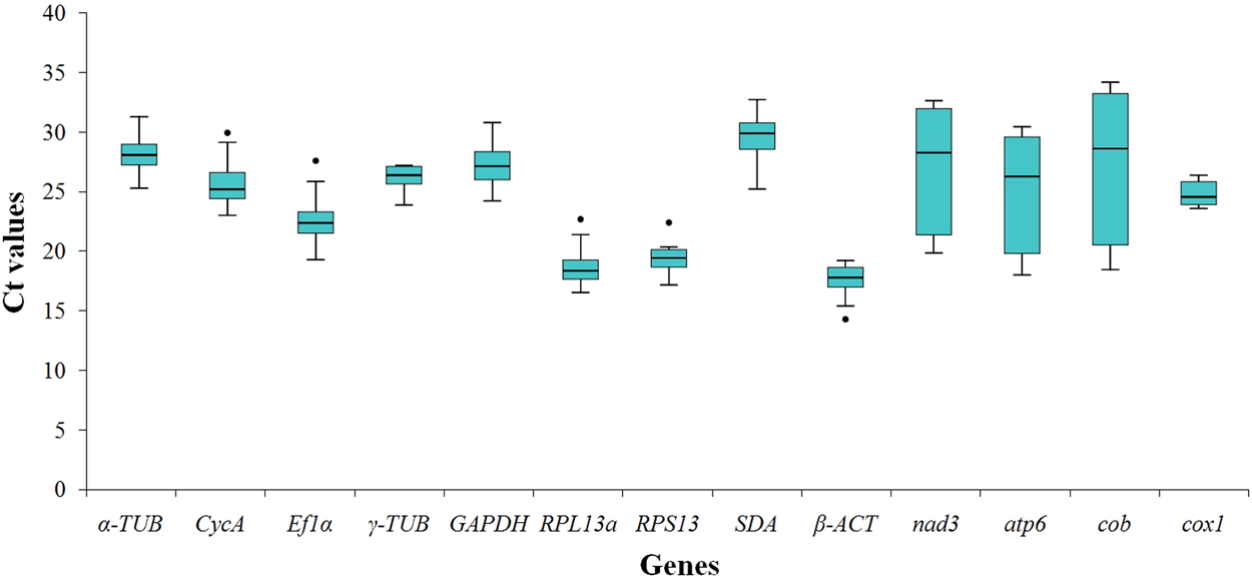
Cycle threshold (Ct) values of the candidate RGs across the experimental samples. The line in the box indicates the median value. The box represents the values between the 25th and 75th percentiles. The maximum and minimum values are represented by up and low caps.

The expression profiles of the nine candidate RGs and four target genes were analyzed by calculating the cycle threshold (Ct) value, which represents the number of cycles required for the amplification to exceed a fixed threshold. As shown in Fig. 1, the distributions of the mean Ct values for each gene across all samples varied significantly. The mean Ct values of the 9 candidate RGs ranged from 17.82 to 29.81, with the lowest and highest Ct values being obtained from *β-ACT* (15.46) and *SDA* (32.85), respectively. Among the target genes, *cox1* was the most abundant transcript with the lowest mean Ct value (24.96) and *cob* was the least abundant mRNA with the highest mean Ct value (27.25).

### Expression stability of the candidate RGs in different developmental stages

**Figure 2:**
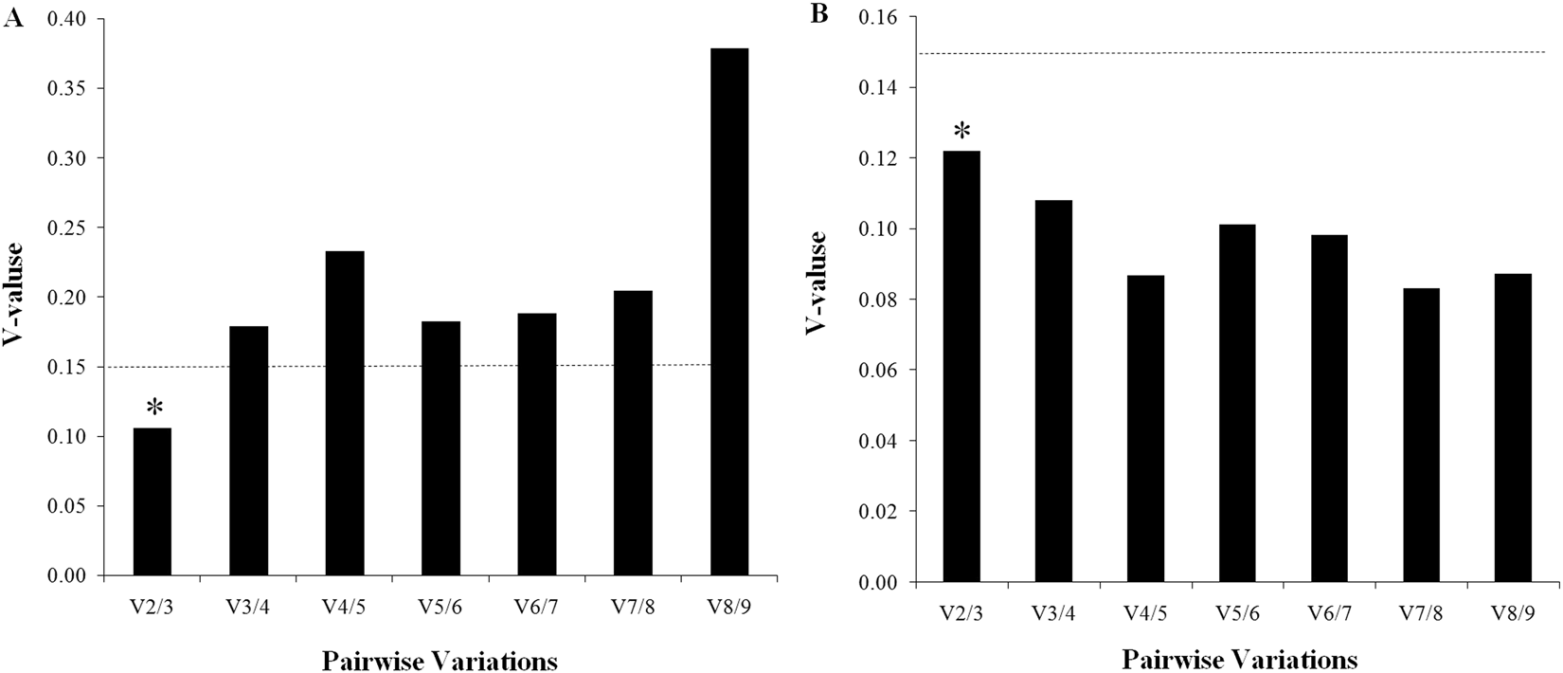
Pairwise variation of RGs in *C. ferrugineus*, according to the geNorm algorithm. Line graphs show the average expression stability value (M) for (**A**) different developmental stages and (**B**) different strains of the insect. Bar graphs show the pairwise variation (V) of normalization factors (NF_n_ and NF_n+1_) to estimate the optimal number of RGs for use in the analysis. The optimum number of RGs required for normalization (as predicted by the algorithm) is marked with an asterisk.

We calculated and ranked the expression stability of nine candidate RGs across the different developmental stages of the insect using four statistical algorithms. The stability varied considerably between the nine candidates according to all four algorithms (Table 2). The most stable RGs recommended by geNorm were largely similar with those identified by the delta Ct method, showing that *RPS13*, *RPL13a* and *EF1α* were the most stable RGs. The three most stable RGs determined by NormFinder were *SDA*, *GAPDH* and *EF1α*, whereas *γ-TUB*, *β-ACT* and *RPS13* were judged to be the most stable by BestKeeper (Table 2). Thus, the different algorithms did not all predict the same RGs as the most stable. However, we found that *RPS13* and *EF1α* were ranked among the top three most stable RGs by at least three algorithms. Interestingly, all four algorithm programs consistently identified *α-TUB* as the least stable RG. The geNorm analysis showed that the pairwise variation value of V_2/3_ (0.106) was below the cut-off value of 0.15 (Fig. 2A), indicating that two RGs are sufficient for accurate normalization across developmental stages. Therefore, it was considered that the two most stable RGs (*RPS13* and *EF1α*) could be normalized across the developmental stages.

**Table 2:** Ranking of RGs based on their expression stability across different developmental stages of *C. ferrugineus* according to geNorm, NormFinder, BestKeeper, and delta Ct method.

Ranking	geNorm (M-value)	NormFinder (stability value)	BestKeeper (SD)	Delta Ct method (stability value)
1	*RPS13*/*RPL13a* (0.288)	*SDA* (0.072)	*γ-TUB* (1.95)	*EF1α* (1.330)
2	—	*GAPDH* (0.270)	*β-ACT* (1.99)	*RPS13* (1.363)
3	*EF1α* (0.327)	*EF1α* (0.531)	*RPS13* (2.09)	*RPL13a* (1.393)
4	*β-ACT* (0.530)	*γ-TUB* (0.540)	*EF1α* (2.12)	*SDA* (1.438)
5	*γ-TUB* (0.796)	*RPS13* (0.635)	*SDA* (2.16)	*GAPDH* (1.474)
6	*SDA* (0.927)	*RPL13a* (0.753)	*RPL13a* (2.22)	*γ-TUB* (1.509)
7	*GAPDH* (1.070)	*CycA* (0.845)	*GAPDH* (2.73)	*β-ACT* (1.587)
8	*CycA* (1.237)	*β-ACT* (1.085)	*CycA* (3.14)	*CycA* (1.749)
9	*α-TUB* (1.730)	*α-TUB* (2.351)	*α-TUB* (3.60)	*α-TUB* (3.456)

### Expression stability of candidate RGs in different phosphine strains

Among the different insect strains tested, the expression stability rankings generated by three of the four algorithms (geNorm, NormFinder, and delta Ct) were highly similar: *RPS13* was identified as the highest expression stability among all the RGs, while *CycA* was the least stable gene (Table 3). The BestKeeper method identified *RPS13* as the most stable of the RGs and *CycA* as one of the least stable genes. *γ-TUB* was identified as the most stable RG by BestKeeper. This gene was also one of the genes with the highest stability according to the NormFinder and delta Ct methods. Taking account of the predictions of all four algorithms, *RPS13* and *γ-TUB* were the most stable RGs. The geNorm analysis revealed that the pairwise variation values of V_2/3_ to V_8/9_ were all below the cut-off value of 0.15 (Fig. 2B). Thus, *RPS13* and *γ-TUB* were the most suitable combination of RGs to use for measuring gene expression in different *C. ferrugineus* strains.

**Table 3:** Ranking of RGs based on their expression stability in different strains according to geNorm, NormFinder, BestKeeper, and delta Ct algorithm methods.

Ranking	geNorm (M-value)	NormFinder (stability value)	BestKeeper(stability value)	Delta Ct method (stability value)
1	*RPS13*/*β-ACT* (0.280)	*RPS13* (0.204)	*γ-TUB* (0.64)	*RPS13* (0.565)
2	—	*γ-TUB* (0.247)	*RPL13a* (0.68)	*β-ACT* (0.600)
3	*EF1α* (0.354)	*β-ACT* (0.260)	*RPS13* (0.66)	*γ-TUB* (0.618)
4	*RPL13a* (0.415)	*α-TUB* (0.262)	*EF1α* (0.73)	*α-TUB* (0.635)
5	*γ-TUB* (0.448)	*EF1α* (0.323)	*β-ACT* (0.77)	*EF1α* (0.656)
6	*α-TUB* (0.519)	*SDA* (0.349)	*α-TUB* (0.88)	*SDA* (0.686)
7	*SDA* (0.586)	*GAPDH* (0.412)	*CycA* (0.93)	*GAPDH* (0.743)
8	*GAPDH* (0.625)	*RPL13a* (0.446)	*SDA* (0.99)	*RPL13a* (0.757)
9	*CycA* (0.683)	*CycA* (0.533)	*GAPDH* (1.04)	*CycA* (0.884)

### Validation of the selected RGs in *C. ferrugineus*

**Figure 3:**
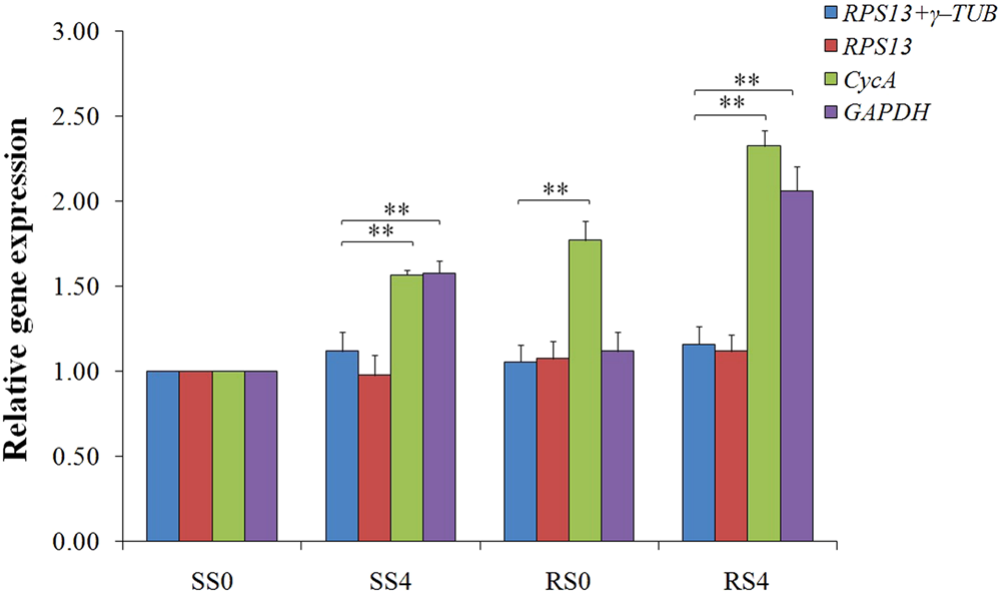
Validation of the selected RGs in *C. ferrugineus*. Expression levels of the target gene (*cox1*) were tested using different normalization factors. The data represent mean values ± SD. Comparison of means is carried out using the Student’s t-test (*P* < 0.05). A double asterisk indicates significantly different values (*P* < 0.01).

To demonstrate the effect of the selected RGs on target gene expressions, the relative expression levels of *cox1* were analyzed using different sets of RGs and compared between the four insect strains, i.e. phosphine-susceptible (SS0), -resistant strains (RS0) and each of these two exposed to phosphine for 4 h (SS4 and RS4) (Table 4). Similar expression profiles were obtained when the expression levels were normalized using the most stable RG (*RPS13*) and the combination of the two most stable RGs (*RPS13* + *γ-TUB*), showing that the transcriptional levels of *cox1* were not significantly different among the different strains (Fig. 3). However, when the expression levels were normalized with the least suitable RG (*CycA*), the expression levels of *cox1* were higher in three strains (SS4, RS0 and RS4) than that in the susceptible strain (SS0). Similarly, the expression levels normalized using another less suitable RG (*GAPDH*) were also higher in the SS4 and RS4 strains than that in the susceptible strain (SS0). When the *cox1* transcript expression levels were normalized by the combination of the two most suitable RGs, they were significantly different from those calculated with the least suitable RG (*CycA* or *GAPDH*), in both the SS4 and RS4 strains (*P* < 0.01) (Fig. 3).

**Table 4:** Sample collection information.

Location and collection time	Site of collection	Susceptibility to phosphine	Sample category	Samples	Individual amount used for RNA extraction
Zhangjiagang County of Jiangsu Province, China, in July, 2013	farmer’s barns	Susceptible strain (SS)	Developmental stages	1st-instar larvae	70
				4th-instar larvae	25
				pupae	25
				newly-emerged adults	22
				aged adults	22
			Duration of exposure to phosphine (h)	0 (SS0)	22
				0.5 (SS0.5)	22
				1 (SS1)	22
				2 (SS2)	22
				4 (SS4)	22
				8 (SS8)	22
				12 (SS12)	22
				48 (SS48)	22
Taicang County of Jiangsu Province, China, in July, 2013	State Grain Reserve Depot	Resistant strain (RS)	Duration of exposure to phosphine (h)	0 (RS0)	22
				0.5 (RS0.5)	22
				1 (RS1)	22
				2 (RS2)	22
				4 (RS4)	22
				8 (RS8)	22
				12 (RS12)	22
				48 (RS48)	22

### Expression profiles of four mitochondrial genes in the different *C. ferrugineus* strains

**Figure 4:**
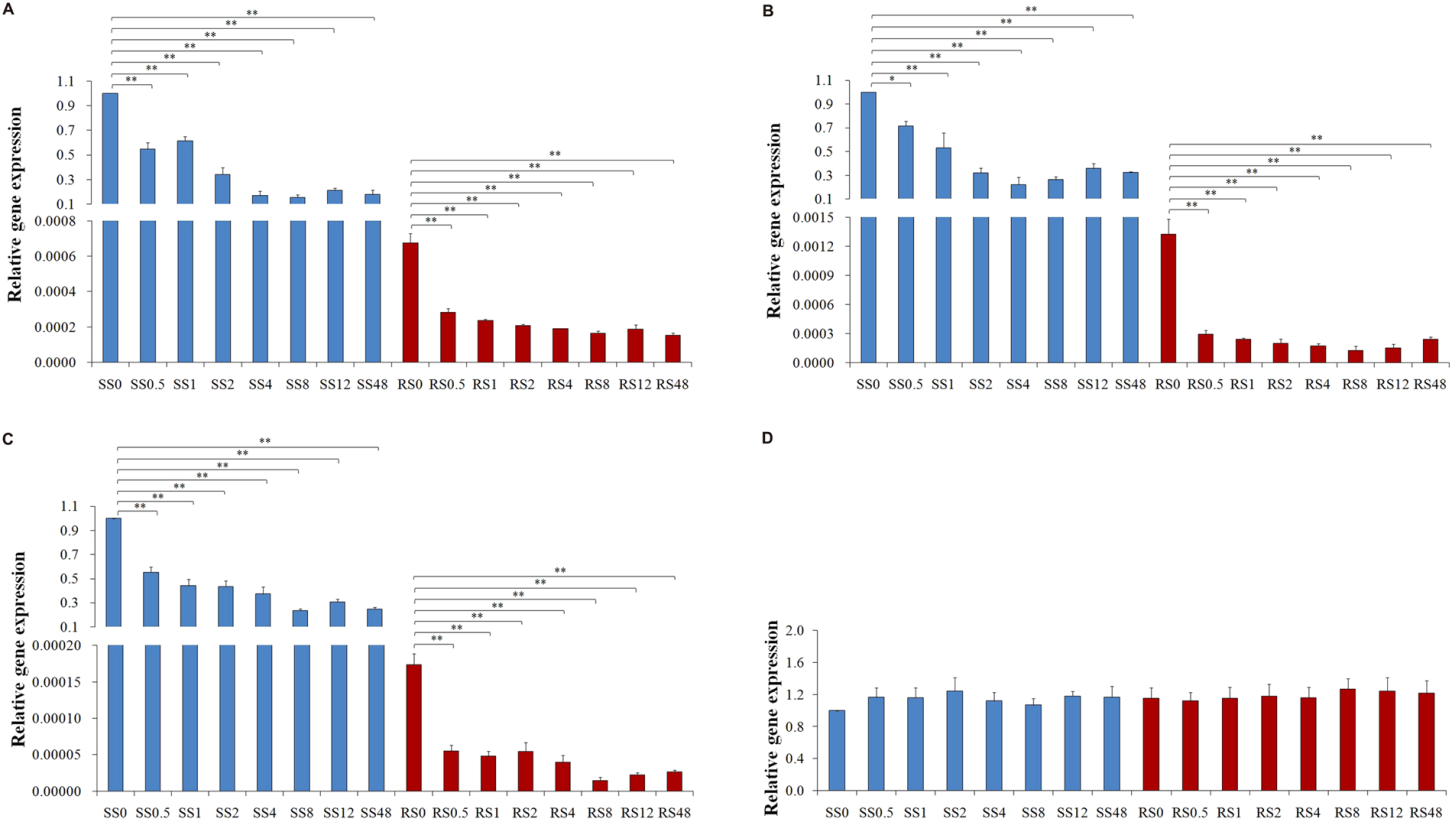
Relative expression of 4 mitochondrial genes according to the selected RGs (*RPS13* + *γ-TUB*). (**A**) *nad3*, (**B**) *atp6*, (**C**) *cob* and (**D**) *cox1* in sample sets across the phosphine-susceptible strain (SS) and phosphine-resistant strain (RS) under exposure to phosphine for 0.5 h, 1 h, 2 h, 4 h, 8 h, 12 h and 48 h, respectively. Bars indicate the standard deviation (±SD) evaluated from three biological replicates. The asterisks indicate a statistically significant difference compared with the control, by the Student’s t-test (SS0 and RS0 were the controls, respectively; *P < 0.05, **P < 0.01).

The relative expression profiles of four mitochondrial genes (*nad3*, *atp6*, *cob* and *cox1*) were assessed in the different *C. ferrugineus* strains using a combination of *RPS13* and *γ-TUB* as the RGs for the normalization of the data (Fig. 4). The results showed that the expression levels of three mitochondrial genes (*nad3*, *atp6* and *cob*) were markedly lower in the phosphine-resistant strains compared to the phosphine-susceptible strains, by approximately 1479, 753, and 5756 fold, respectively (Fig. 4A–C). When exposed to phosphine, the transcriptional expression levels of these three genes were significantly lower than those in the unexposed strains, regardless of the duration of exposure (*P* < 0.01), and there was an overall decreasing trend in expression levels with increasing duration of exposure in all strains. However, the extent of this decline for any given exposure duration was different between the susceptible and resistant strains, especially for the shorter exposure times (0.5 h, 1 h and 2 h). After 0.5 h exposure to phosphine, the expression levels of *cob* had decreased from 1 (RS0) to 0.32 (RS0.5) in the resistant strain, but had decreased by only 0.55 times (SS0.5/SS0) in the susceptible strain (Fig. 4C). Clearly, the resistant strains responded faster to conditions of phosphine stressors than the susceptible strains. For *cox1* under the same exposure duration, the expression levels changed from 1.07- (SS8) to 1.27-times (RS8) in the resistant strains, but there was no significant difference among any of all the strains in this respect (*P* > 0.05) (Fig. 4D).

## Discussion

In this study, we performed a comprehensive analysis of nine RGs in *C. ferrugineus* to aid in the normalization of gene expression analyses by qRT-PCR since qRT-PCR has proven effective for exploring the underlying mechanisms of insecticide resistance in many insect pests^44–46^. We found that the most stable RGs across different developmental stages differed among different algorithms, whereas *α-TUB* was consistently identified as the least stable RG by all four algorithms. *SDA* and *GAPDH* were found to be the most stable RGs by NormFinder; however, they were not determined as stable by the other three algorithms. For different insect strains, slight differences in the stability ranking were observed among the four algorithms. *RPS13*, *γ-TUB*, *β-ACT* and *EF1α* were identified as the top four most stable RGs across strains, whereas *CycA* and *GAPDH* were ranked as the two least stable RGs, by all four algorithms. These variations have also been obtained in many previous studies of insects, such as *Lipaphis erysimi* ^30^, *Galeruca daurica* ^28^, *Aphis gossypii* ^37^ and *Helicoverpa armigera* ^29^, as different algorithms and analytical procedures are used in the four algorithm programmes and these do not seem to affect the overall validation quality^47^.

*RPS13* has been identified as the most stable gene in different tissues of *Sesamia inferens* ^48^, and *RPS13* mRNA expression was shown to be unaffected by alcohol administration, SIV infection, or antiviral therapy in rhesus macaques^49^. Our results demonstrated that *RPS13* was the most stable gene both among different developmental stages and among different strains of *C. ferrugineus*, indicating that *RPS13* exhibits a wide degree of stability in a range of conditions. *EF1α* is a commonly used housekeeping gene in qRT-PCR experiments^50^. Previously, *EF1α* has been reported to exhibit a stable level of expression in *A. gossypii* under biotic and abiotic stressors^37^. Being an important specific protein factor involved in the process of protein translation, it was not suprising that *EF1α* was identified in the present study as one of the most stable RGs in terms of its expression across the developmental stages of *C. ferrugineus*. However, it was not the most stable RG among different phosphine strains of *C. ferrugineus*. *γ-TUB*, a gene that has not previously been identified as a stable RG, was determined to be one of the most stable RGs across different strains in the present study, but it was not the most appropriate RG in terms of its stability across different developmental stages in *C. ferrugineus*.

*GAPDH* has previously been confirmed to be one of the most suitable RGs in a number of other studies^35–37^, although low *GAPDH* expression stablitity has been observed in a few studies under certain conditions, such as in different tissues, developmental stages and treatments of *Agrilus planipennis* ^50^. In another study, *GAPDH* was found to be the least stable gene in phosphine-induced strains of *T. castaneum* ^51^. In the present study, we identified *GAPDH* as an unsuitable RG across different strains in *C. ferrugineus* ^51^. *RPL13a* was calculated as one of the most stable RGs to phosphine induction in *T. castaneum* ^51^, but it did not exhibit such a high degree of stability across different phosphine strains of *C. ferrugineus* in the present study, indicating that the stability of the same RG may be species-specific. *α-TUB* has also been widely used as a RG in gene expression studies in *G. daurica* ^28^ and *Sogatella furcifera* ^36^. However, other researcher have revealed that *α-TUB* does not satisfy certain basic requirements for use as an internal control^37^. In the present study, the results showed that the expression levels of *α-TUB* were not stable across different developmental stages of *C. ferrugineus*, therefore this gene would not be the most reliable internal control for comparative gene expression analyses. These differences may partly be explained by the fact that α/β-*TUB* not only acts as one of the major components of the cytoskeletal structure of cells, but also participates in other cellular functions^52^. The structural protein *β-ACT* is expressed at moderately abundant levels in most cell types and has been considered an ideal RG for measuring gene expressions in many insects, including *Drosophila melanogaster* ^34^ and *Apis mellifera* ^53^. However, *β-ACT* was not the most stable gene in the present study, which is consistent with some previous studies on *A. planipennis* ^50^ and *Frankiniella occidentails* ^32^. These differences highlighted the need to validate RGs for different species, even under similar experimental conditions.

Respiratory complexes I, III and IV constitute the main electron transport chain in aerobic organisms and generate the proton gradient in the intermembrane spaces of mitochondria. Complex V, or ATP synthase, uses the energy stored in a proton gradient across a membrane to drive the synthesis of ATP from ADP and phosphate (Pi)^54^. In the present study, the transcriptional profiles of four mitochondrial genes (*nad3*, *cob*, *cox1* and *atp6*), encoding the subunits of complexes I, III, IV and V, respectively), were different in various phosphine strains of *C. ferrugineus*, indicating that these subunits may have different roles in protecting against phosphine stressors. No significant expression differences were observed in *cox1* among the phosphine strains in the present study even though Complex IV has previously been proposed as the principal mitochondrial site of action of phosphine^20, 22, 55^. However, a study on *cox1* and *cox2* expressions in *S. zeamais* found no significant association with phosphine susceptibility, in agreement with our findings^56^. Our results suggested that modifications in the expressions of the *cox1* gene encoding complex IV may be not associated with phosphine resistance in *C. ferrugineus*. In contrast, three other mitochondrial genes (*nad3*, *cob* and *atp6*) were significantly suppressed in *C. ferrugineus* strains exposed to phosphine, and showed extremely low expressions in the phosphine-resistant strain compared to the phosphine-susceptible strain. This could indicate that phosphine may inhibit the insect respiratory system and that a lower respiratory rate may be an important mechanism for phosphine resistance in *C. ferrugineus*. Our results in this respect are largely in agreement with those of previous studies conducted in *T. castaneum* ^12^, *R. dominica* ^12^, *O. surinamensis* ^12^, *S. zeamais* ^55^, *Musca domestica* ^22^ and *Sitophilus granaries* ^22^. Previous studies also showed that the suppression of mitochondrial respiratory chain genes caused an increase in phosphine resistance in *Caenorhabditis elegans*, and the strongest resistance towards phosphine was identified when the complex III genes were suppressed^57^. These results could explain our observations regarding the magnitude of the decrease in the expression of *cob* (5756 fold), compared to that of *nad3* (1479 fold) and *atp6* (753 fold), suggesting that complex III may play a more important role in phosphine resistance in *C. ferrugineus*. Additionally, we found that when *C. ferrugineus* were exposed to phosphine, the transcriptional expression levels of *nad3*, *atp6* and *cob* were altered more significantly in the phosphine-resistant strain than in the phosphine-susceptible strain, indicating a difference in the response to phosphine between the two strains. A compensation mechanism of ATP synthesis should exist in phosphine resistant insects, considering that no fitness cost has been reported in phosphine-resistant strains of the red flour beetle (*T. castaneum*)^10, 58^.

Overall, our results have shown that expression levels in the mitochondrial genes encoded by mitochondria were negatively related to phosphine resistance in *C. ferrugineus*, supporting the existing hypothesis that phosphine directly targets the mitochondria. There were marked differences in the expression levels of mitochondrial genes between the susceptible and resistant strains of *C. ferrugineus*.

## Methods

### Insect sampling and rearing

#### Insect rearing

Phosphine-susceptible strain of *C. ferrugineus* was originally collected from farmer’s barns in Zhangjiagang County, Suzhou City, Jiangsu Province, China, in July 2013 (LC_50_ = 0.035 mg/L, SS). Phosphine-resistant strain was originally collected from the State Grain Reserve Depot in Taicang County, Suzhou City, Jiangsu Province, China, in July 2013 (LC_50_ = 18.76 mg/L, RS). Both strains have been maintained in our laboratory since collection, without any special methods to maintain the resistance levels. When performing the present study in 2016, we also measured the LC_50_ values for these two strains and found that the LC_50_ values almost unchanged compared to that in 2013. All the insects used in this study were reared on whole wheat flour and yeast powder (10:1) in a growth chamber under conditions of 32 ± 1 °C, and 70–80% relative humidity, in total darkness.

### Experimental treatments

#### Developmental stages

Five different developmental stages, first instar larvae, fourth instar larvae, pupae, newly emerged adults and aged adults were collected from the susceptible strain (Table 4). Hundreds of adults (of both sexes) were released on to a culture dish containing a small amount of feed, allowed to lay eggs and removed after 48 h. First instar larvae, fourth instar larvae and pupae were collected after approximately 6, 20 and 26 days, respectively. A proportion of the pupae were transferred to a new culture dish, from which newly emerged adults were collected after approximately 8 days, and aged adults were collected after approximately 40 days. Enough insect samples were collected for three biological replicates and these were immediately frozen in liquid nitrogen and then stored at −80 °C for RNA extraction.

#### Phosphine-induced stress

Two-week old adults of both susceptible and resistant strains of *C. ferrugineus* were exposed to phosphine (0.026 mg/L, LC_30_ of the susceptible strain) in sealed gas-tight glass jars with a 1 L volume at 30 °C. The control groups were treated with an equal volume of air. The surviving insects were collected after 0.5, 1, 2, 4, 8, 12 and 48 h, respectively (Table 4). Enough samples were collected to provide three biological replicates and these were immediately frozen in liquid nitrogen and stored at −80 °C until used.

### Total RNA isolation and cDNA synthesis

Total RNA was isolated by the RNeasy Plus Micro Kit (Qiagen, Germany) following the manufacturer’s instructions. Genomic DNA was removed using a genomic DNA elimination column supplied with the kit. The purity and concentration of total RNA were assessed with a BioMate 3 S (Thermo Scientific, USA). RNA samples with an OD_260_/OD_280_ ratio ranging from 1.8 to 2.2 and an OD_260_/OD_230_ ratio >2.0 were stored at −80 °C for further processing. First-strand cDNA synthesis was carried out with 1 μg total RNA in a 20 μl reaction volume using a PrimeScript^Tm^II 1st Strand cDNA Synthesis Kit (Takara, Japan). cDNA was stored at −20 °C until use. Each cDNA sample was diluted 10 times with nuclease free water before being used as a template for qRT-PCR.

### Selection of reference genes and primer design

We selected nine candidate RGs to investigate their stability as internal controls for qRT-PCR (Table 1). The sequences of the selected RGs were obtained from the *C. ferrugineus* transcriptome sequence (accession number: SRA245468). Four target genes (*nad3*, *atp6*, *cob* and *cox1*), obtained from the mitochondrial genome of *C. ferrugineus* (unpublished data), were selected to determine their phosphine resistance status. The primers used in qRT-PCR were designed using the online primer design software Primer 3.0 (http://frodo.wi.mit.edu/primer3/).

### Quantitative real-time PCR and amplification efficiency test

qRT-PCR was performed using an ABI 7500 PCR system (Applied Biosystems, USA). The 25 μl reactions contained 1 μl of cDNA template, 12.5 μl of QuantiFast^®^SYBR^®^ Green PCR Master Mix (Qiagen, Germany) and 0.5 μl of each primer. Nuclease-free water was added to make the volume up to 25 μl. Thermal cycling conditions were 95 °C for 5 min followed by 40 cycles of 95 °C for 10 sec followed by 60 °C for 30 sec. At the end of each PCR run, a melting curve analysis from 55 to 95 °C was applied to all reactions to ensure the specificity of the amplified product. Each qRT-PCR reaction was performed in triplicate (technical replicates) on three individual samples (biological replicates). Standard curves were constructed to determine PCR efficiency (E%) and correlation co-efficient (*R* ^2^) based on a 5-fold dilution series of cDNA. The PCR efficiency (E%) was calculated according to the equation: E = (10^[−1/slope]^ − 1) × 100^59^.

### Expression stability analysis

All biological replicates were used to calculate the average Ct value. Stability values of the nine candidate RGs were assessed using four commonly used software tools: geNorm version 3.5^39^, NormFinder version 0.953^40^, BestKeeper^41^ and delta Ct^42^, which utilize different algorithms to calculate the stability in expression levels across samples.

The geNorm program estimates the stability of each gene by calculating an expression stability value “M” and ranks them in an order for a given set of samples. Values of M that are less than 1.5 are considered to identify a stable level of gene expression across treatments. The lower the M value, the more stable the expression. Furthermore, geNorm provides an algorithm to calculate the optimal number of RGs required for better normalization, because use of a single RG may result in inaccurate normalization, leading to significant errors in gene expression analyses^39^. The pairwise variation (V) of these genes is compared with that of others, and the pairwise variation values (V_n_/V_n+1_) between two sequential normalization factors were used to determine the optimal number of RGs. A pairwise variation value of less than 0.15 implies that the addition of another RG will not make any significant contribution to the accuracy of normalization^39^.

NormFinder calculates the gene expression stability values by taking into account variations between and within defined sample groups or treatments, and provides a gene rank order depending on the values obtained. The candidate gene with the lowest value is considered to be the most stable RG^40^. BestKeeper determines the best-suited RGs based on the geometric mean of the Ct values and PCR amplification efficiency, and combines them into an index^41^. The comparative delta Ct method compares basic Ct values and the relative expression of ‘gene pairs’ within each sample, to confidently identify reliable RGs^42^.

### Validation of reference gene selection

To evaluate the validity of the optimized selection of RGs, the expression levels of one target gene (*cox1*) were analyzed in 4 different *C. ferrugineus* strains, i.e. phosphine-susceptible (SS0), phosphine-resistant (RS0) and each of these two exposed to phosphine for 4 h (SS4 and RS4). The least stable genes were *CycA* (as determined by geNorm, NormFinder and delta Ct method) and *GAPDH* (as determined by BestKeeper). The most stable gene was *RPS13* (as determined by geNorm, NormFinder and delta Ct method) and the combined set of RGs *RPS13* + *γ-TUB* (as calculated and recommended from the findings of this study) were used for comparative analyses. The relative expression levels were calculated according to the 2^−ΔΔCT^ method^60^. The target gene expression was normalized by only one RG (*CycA*, *GAPDH* or *RPS13*), and the recommended combination of RGs were calculated using a Student’s t-test, in SPSS 17.0 software, with significance levels denoted by *(0.01 < *P* < 0.05) and **(*P* < 0.01). All experiments were performed in triplicate, and the results were expressed as means ± standard deviation (SD).

### Determination of the expression profiles of selected mitochondrial genes

The expression profiles of *nad3*, *atp6*, *cob* and *cox1* were calculated in different strains, comprising both susceptible and resistant strains exposed to phosphine for 0.5, 1, 2, 4, 8, 12, 48 h in a concentration of 0.026 mg/L (LC_30_ of susceptible strain), with the combined set of RGs (*RPS13* + *γ-TUB*). The relative expression of the four target genes was calculated using the 2^−ΔΔCt^ method^60^. All the experiments were performed in triplicate, and the results were expressed as means ± SD. The target gene expression of phosphine exposed and unexposed strains were calculated using a Student’s t-test, in SPSS 17.0 software, with significance levels denoted by * (0.01 < *P* < 0.05) and ** (*P* < 0.01).

## Conclusions

This study evaluated the stability of expression of nine RGs for normalization in qRT-PCR of *C. ferrugineus* using four algorithms, and then explored whether the differences in expression of four mitochondrial genes were associated with phosphine resistance in this pest. Our results showed that *RPS13* and *EF1α* were the most stable RGs across different *C. ferrugineus* stages, whereas *RPS13* and *γ-TUB* were the most stable across the different insect strains. Expression changes in mitochondrial genes may be an important mechanism of phosphine resistance in *C. ferrugineus*. Our results will benefit future functional genomics studies of the development and phosphine resistance in *C. ferrugineus*.

## Electronic supplementary material

Supplementary information
