# Supplementary material for: Reference gene selection to determine differences in mitochondrial gene expressions in phosphine-susceptible and phosphine-resistant strains of Cryptolestes ferrugineus, using qRT-PCR
Source: Sci Rep. 2017 Aug 1;7:7047. doi: 10.1038/s41598-017-07430-2 (PMC5539111; doi:10.1038/s41598-017-07430-2)
Supplement: Supplementary file 1 — Supplementary information [file 41598_2017_7430_MOESM1_ESM.pdf]

Supplementary Information

Title: Reference gene selection to determine differences in mitochondrial gene expressions in phosphine-susceptible and phosphine-resistant strains of *Cryptolestes ferrugineus*, using qRT-PCR

Author list: Pei-An Tang[1, *], Jin-Yan Duan[1], Hai Jing Wu[1], Xiong-Rong Ju[1], Ming-Long Yuan[2, *]

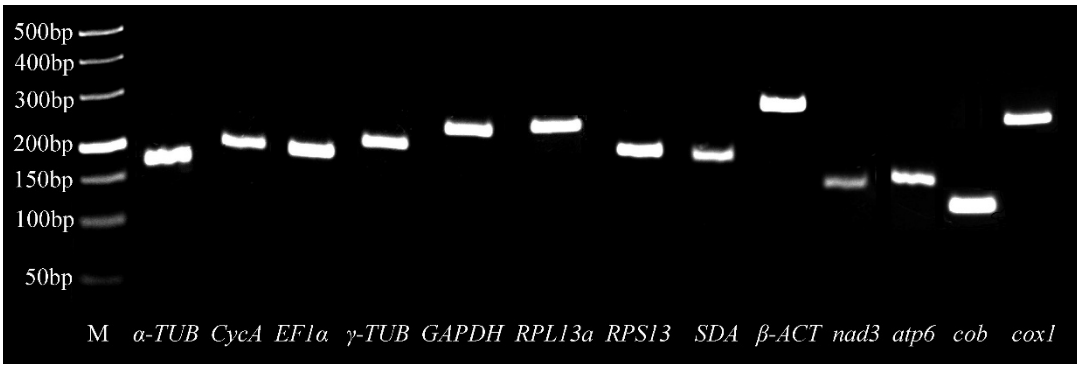

Supplementary Figure S1. 3% agarose gel electrophoresis is exhibiting specific qRT-PCR products with expected size for each candidate reference gene. M, DNA marker (Takara, DL 500); *α–TUB, α-tubulin*; *CycA, cyclinA*; *EF1α, elongation factor 1α*; *γ–TUB, γ-tubulin*; *GAPDH, glycerladehyde-3-phosphate dehydrogenase*; *RPL13a, ribosomal protein S13a*; *RPS13, ribosomal protein S13*; *SDA, succinate dehydrogenase complex subunit A*; *β-ACT, beta actin*; *nad3, NADH dehydrogenase subunit 3*; *atp6, ATP synthase F0 subunit 6*; *cob, cytochrome b*; *cox1, cytochrome c oxidase subunit I.*

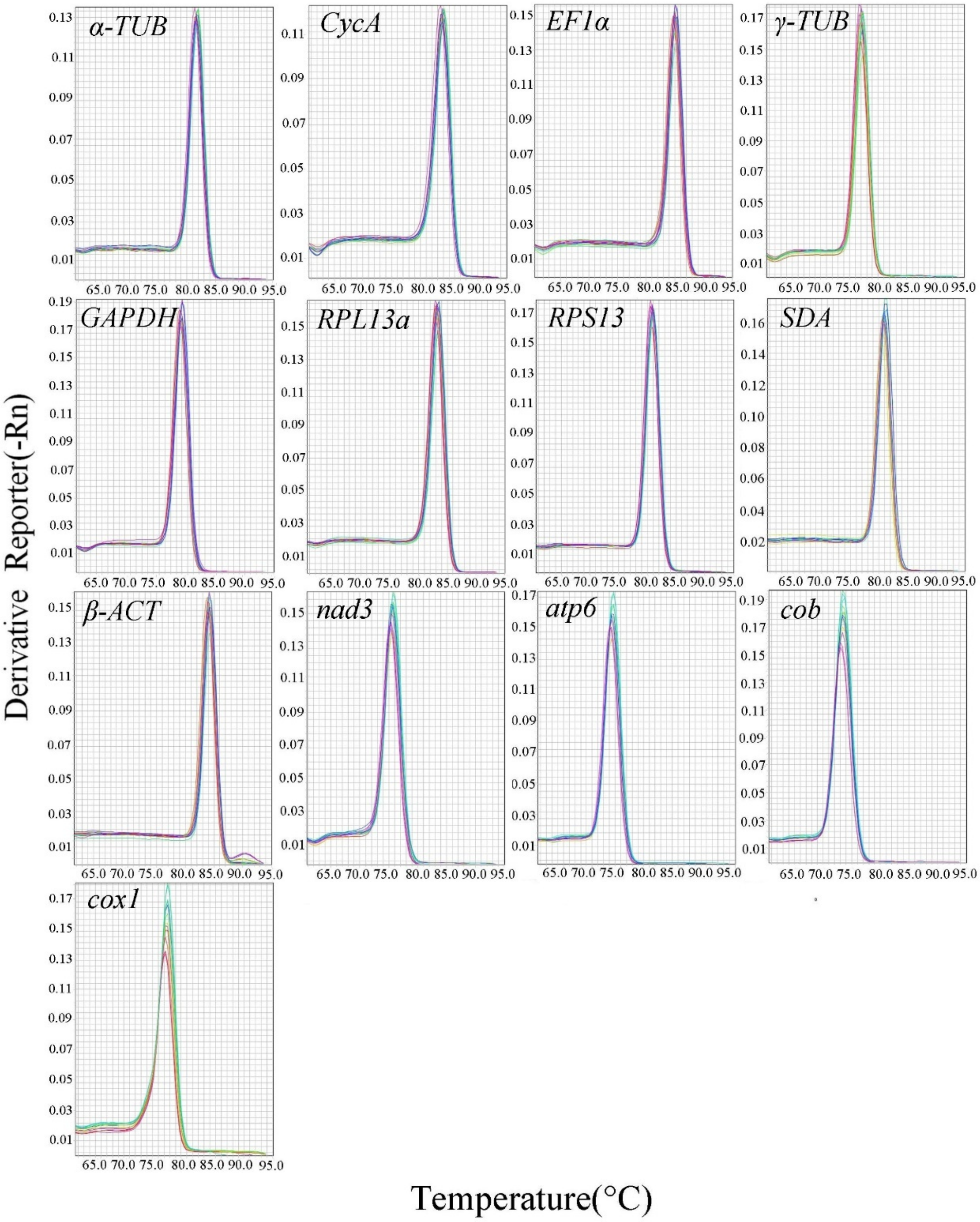

Supplementary Figure S2. **Dissociation curves of nine candidate reference genes and four target genes reveal single peaks.** *α–TUB, α-tubulin*; *CycA, cyclinA*; *EF1α, elongation factor 1α*; *γ–TUB, γ-tubulin*; *GAPDH, glycerladehyde-3-phosphate dehydrogenase*; *RPL13a, ribosomal protein S13a*; *RPS13, ribosomal protein S13*; *SDA, succinate dehydrogenase complex subunit A*; *β-ACT, beta actin*; *nad3, NADH dehydrogenase subunit 3*; *atp6, ATP synthase F0 subunit 6*; *cob, cytochrome b*; *cox1, cytochrome c oxidase subunit I*. The results were obtained from three technical replicates of different cDNA samples along with no template control.

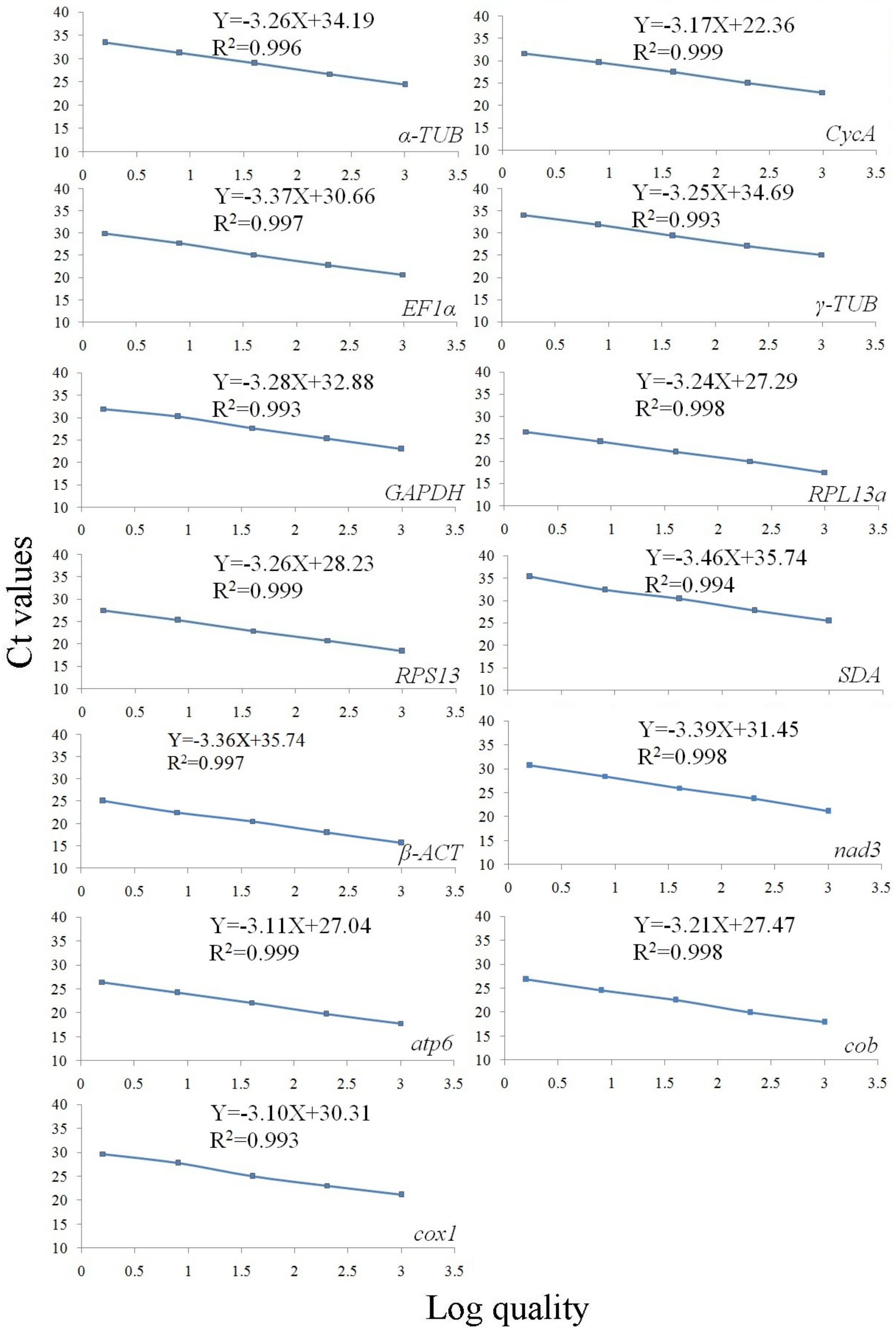

Supplementary Figure S3. **Standard curves of nine candidate reference genes and four target genes**. Line relations and correlation coefficient (R^2) between Ct value and Log quantity were calculated by SigmaPlot 12.0 software.
